# I-131 Dose Response for Incident Thyroid Cancers in Ukraine Related to the Chornobyl Accident

**DOI:** 10.1289/ehp.1002674

**Published:** 2011-03-17

**Authors:** Alina V. Brenner, Mykola D. Tronko, Maureen Hatch, Tetyana I. Bogdanova, Valery A. Oliynik, Jay H. Lubin, Lydia B. Zablotska, Valery P. Tereschenko, Robert J. McConnell, Galina A. Zamotaeva, Patrick O’Kane, Andre C. Bouville, Ludmila V. Chaykovskaya, Ellen Greenebaum, Ihor P. Paster, Victor M. Shpak, Elaine Ron

**Affiliations:** 1 Division of Cancer Epidemiology and Genetics, National Cancer Institute, National Institutes of Health, Department of Health and Human Services, Bethesda, Maryland, USA; 2 Institute of Endocrinology and Metabolism, Kyiv, Ukraine; 3 Department of Epidemiology and Biostatistics, University of California–San Francisco, San Francisco, California, USA; 4 Department of Medicine, The Thyroid Clinic, College of Physicians and Surgeons, Columbia University, New York, New York, USA; 5 Department of Radiology, Thomas Jefferson University Hospital, Philadelphia, Pennsylvania, USA; 6 Department of Pathology, College of Physicians and Surgeons, Columbia University, New York, New York, USA

**Keywords:** Chernobyl nuclear accident, Chornobyl, Ukraine, 1986, dose–response relationship, incidence, thyroid neoplasms/epidemiology, iodine, radioactive, radiation

## Abstract

Background: Current knowledge about Chornobyl-related thyroid cancer risks comes from ecological studies based on grouped doses, case–control studies, and studies of prevalent cancers.

Objective: To address this limitation, we evaluated the dose–response relationship for incident thyroid cancers using measurement-based individual iodine-131 (I-131) thyroid dose estimates in a prospective analytic cohort study.

Methods: The cohort consists of individuals < 18 years of age on 26 April 1986 who resided in three contaminated oblasts (states) of Ukraine and underwent up to four thyroid screening examinations between 1998 and 2007 (*n* = 12,514). Thyroid doses of I-131 were estimated based on individual radioactivity measurements taken within 2 months after the accident, environmental transport models, and interview data. Excess radiation risks were estimated using Poisson regression models.

Results: Sixty-five incident thyroid cancers were diagnosed during the second through fourth screenings and 73,004 person-years (PY) of observation. The dose–response relationship was consistent with linearity on relative and absolute scales, although the excess relative risk (ERR) model described data better than did the excess absolute risk (EAR) model. The ERR per gray was 1.91 [95% confidence interval (CI), 0.43–6.34], and the EAR per 10^4^ PY/Gy was 2.21 (95% CI, 0.04–5.78). The ERR per gray varied significantly by oblast of residence but not by time since exposure, use of iodine prophylaxis, iodine status, sex, age, or tumor size.

Conclusions: I-131–related thyroid cancer risks persisted for two decades after exposure, with no evidence of decrease during the observation period. The radiation risks, although smaller, are compatible with those of retrospective and ecological post-Chornobyl studies.

Studies conducted approximately a decade after the Chornobyl nuclear power plant accident among individuals exposed as children or adolescents have demonstrated strong, consistent associations between radiation dose and risk of thyroid cancer with estimated odds ratios of approximately 5 at 1 Gy of exposure (Cardis et al. 2005; Davis et al. 2004; Ron 2007; Tronko et al. 2006a). The estimates of relative risk (RR) per gray from ecological studies are even higher (Jacob et al. 1999, 2006; Likhtarov et al. 2006). Ecological studies, however, are subject to limitations because of their use of grouped rather than individual doses and limited data on confounding factors (Lubin 2002). Although there has been substantial progress in quantifying iodine-131 (I-131)-related thyroid cancer risk, prospective incidence data from analytic epidemiological studies are lacking. Because thyroid cancers attributed to I-131 exposure during childhood, similar to external irradiation, continue to occur throughout adulthood (Ron et al. 1995), it is important to evaluate patterns of excess absolute and RRs over time. Also, in the view of mild to moderate iodine deficiency in northern Ukraine (Ashizawa et al. 1997; Bolshova et al. 1993; Tronko et al. 2005), additional data are necessary to clarify the potential modifying effect of iodine prophylaxis and/or stable iodine intake on I-131–related risk of thyroid cancer (Cardis et al. 2005; Shakhtarin et al. 2003).

In the present study, we examined several unresolved issues using prospective data from a cohort composed of approximately 12,500 individuals who were < 18 years of age when the accident occurred and had individual radioactivity measurements taken within 2 months after the accident (Stezhko et al. 2004). The relationship between dose and prevalent thyroid cancers diagnosed during the first (baseline) screening examination (1998–2000) has been reported (Tronko et al. 2006a). The major objective of the present study was to evaluate the dose–response relationship for incident thyroid cancers diagnosed as a result of second to fourth screening examinations based on up to 9 years of follow-up.

## Materials and Methods

*The cohort.* Details of the study design have been published previously (Stezhko et al. 2004; Tronko et al. 2006a). In brief, the cohort includes individuals with direct thyroid radioactivity measurements made in May or June 1986 who were < 18 years of age on 26 April 1986 and resided in selected areas in the neighboring Chernihiv, Zhytomyr, or Kyiv oblasts of Ukraine in 1998. An oblast is an administrative subdivision similar in size to a state or province. Of 32,385 individuals originally selected for the study, 10,307 (31.8%) could not be traced primarily because of the long interval between the accident and the start of screening, as well as high mobility of this young cohort; 2,466 (7.6%) were traced but were not eligible or available to participate; and 6,369 (19.7%) were traced but refused to participate or failed to attend the screening, resulting in 13,243 (40.9%) individuals who were screened for the first time between 1998 and 2000. After additional exclusions described elsewhere (Tronko et al. 2006a), analysis of thyroid cancer prevalence was based on 13,127 individuals. In the present analysis, we also excluded 45 individuals who were diagnosed with thyroid cancer as the result of the first screening examination, 3 individuals who were found to have thyroid aplasia, and 566 individuals (4.3%) who were considered lost to follow-up because they participated in only the first screening examination. We included 1 individual who was diagnosed with incident thyroid cancer 8 years after the first examination but was previously excluded from the analyses because of incomplete data at baseline. This resulted in a total of 12,514 individuals included in the present analysis.

The study was reviewed and approved by the institutional review boards of the participating organizations in Ukraine and the United States, and all participants (or their legal guardians for those < 16 years of age at the time of screening) signed an informed consent form.

*Screening examination.* After a first screening examination in 1998–2000, three biennial thyroid examinations were conducted between 2001 and May 2007 either by a mobile screening team or at the Institute of Endocrinology and Metabolism (IEM) in Kyiv. Screening procedures were standardized and included thyroid palpation and ultrasonographic examination by a trained ultrasonographer; independent clinical examination and palpation by an endocrinologist; a serum sample; a spot urine sample; and a series of structured questionnaires eliciting information on demographics, medical history, and items relevant to thyroid dose estimation, such as residential history, milk consumption, and iodine prophylaxis in May–June 1986.

*Ultrasound examination.* Before 2004, the thyroid gland was examined using 7.5 MHz probes, either an electronic linear transducer (Hitachi Medical Systems, Tokyo, Japan; or GE Logiq 100, General Electric Company, Milwaukee, WI, USA) or a mechanical sector probe with water bag kit (Tosbee SSA 240s with 7.5 MHz SM-708A probes; Toshiba Corp., Tokyo, Japan). Beginning in 2004, this equipment was replaced with a laptop-based mobile system that used a 10-MHz linear probe (Terason Ultrasound, Burlington, MA, USA). Details of nodules, echostructure, and echogenicity were recorded. The thyroid volume was calculated based on the volume of an ellipsoid as described by Brunn et al. (1981).

*Serum assays.* Antibodies to thyroid peroxidase (ATPO), thyroid-stimulating hormone (TSH), and thyroglobulin (TG) were measured in all available serum samples (99% of the cohort) with LUMitest immunochemiluminescence assays (BRAHMS Diagnostica GMBH, Heningsdorf, Germany) using an AutoLumat LB 953 Luminometer (Berthold, Pforzheim, Germany). Based on evaluation of reference limits in a reference sample from our cohort, an elevation of ATPO > 60 U/mL, consistent with BRAHMS, was considered positive. Similarly, based on evaluation of reference limits in a sample from our cohort, reference limits of TSH were set between 0.3 mIU/L and 4.0 mIU/L. According to the manufacturer, reference limits of TG measurements were between 2 ng/mL and 70 ng/mL.

*Iodine determination.* Urinary iodine content was measured using the Sandell-Kolthoff reaction (Dunn et al. 1993) as described previously (Tronko et al. 2005).

*Thyroid cancer cases.* Incident thyroid cancers were defined as histologically confirmed cancers that were first suspected as a result of clinical and laboratory findings during the second to fourth screening examinations (2001–2007) and for which surgery was performed by December 2008. All individuals with a nodule of ≥ 10 mm in its largest dimension, or a nodule of 5–10 mm with ultrasound characteristics suggestive of malignancy (Stezhko et al. 2004), were referred for fine-needle aspiration biopsy (FNAB) at the IEM. If an individual’s FNAB findings were diagnostic or suspicious for malignancy or follicular neoplasia, the person was referred for thyroid surgery. Of 855 individuals referred for FNAB during the second to fourth examinations, 637 (75%) complied, 38 (4%) did not comply, and for 180 (21%) the referral was canceled after a repeated ultrasound examination. Of the 109 individuals referred for thyroid surgery, 87 (80%) complied by December 2008. The International Pathology Panel, established in the framework of the Chernobyl Tissue Bank (2010), reviewed all histopathological diagnoses and confirmed all 65 cancers: 61 papillary (with one incidentally found microcarcinoma), 3 follicular, and 1 medullary thyroid cancer.

*Dosimetry.* Dosimetric methods have been described elsewhere (Likhtarev et al. 2003, 2006; Likhtarov et al. 2005). Briefly, individual I-131 thyroid doses and their uncertainties were estimated from the combination of thyroid radioactivity measurements, data on dietary and lifestyle habits, and environmental transfer models using a Monte Carlo procedure with 1,000 realizations per individual (Likhtarev et al. 2003). The distribution of 1,000 individual I-131 dose estimates was close to lognormal, with geometric SDs ranging from 1.6 to 5.0 for most cohort members (Likhtarev et al. 2003). For the analysis, we used the arithmetic mean of each individual’s 1,000 realizations as the best estimate of I-131 dose. Because these dose estimates were derived from thyroid masses typical of iodine-sufficient populations (International Commission on Radiological Protection 1989), we applied a correction coefficient to adjust them for thyroid masses typical of the Ukrainian population using data collected by the Sasakawa Memorial Health Foundation (for children 5–16 years of age) (Nikiforova et al. 1997) and by the Ukrainian Radiation Protection Institute (for children < 5 years of age) (Likhtarev IA, personal communication). In the analysis cohort, the arithmetic means [geometric means (GMs)] of individual I-131 arithmetic means adjusted for thyroid mass typical of this region were 0.65 (0.20) Gy compared with the original means of 0.77 (0.26) Gy, respectively (Tronko et al. 2006a). In general, dose estimates were negatively correlated with age at time of the accident. Individual thyroid dose estimates currently are available only for I-131 and not for other isotopes of iodine or cesium (Stezhko et al. 2004). However, I-131 typically accounts for 90–95% of total thyroid dose (Likhtarev et al. 2003; Stezhko et al. 2004).

*Statistical analysis.* We counted person-years (PY) at risk from the date of the first screening examination to the date of thyroid surgery or to the date of last screening examination for those not operated upon. Data were cross-classified by age at exposure (from 0 to 18 years in 2-year intervals), attained age (from 12 to 40 years by 2-year intervals), dose estimates (< 0.05, 0.05–0.09, 0.1–0.29, 0.3–0.49, 0.5–0.69, 0.7–0.99, 1.0–1.49, 1.5–1.99, 2.0–2.49, 2.5–2.99, ≥ 3.0 Gy), and calendar time intervals (1998–2008 in 1-year intervals).

In addition, data were cross-classified by the following categorical variables reflecting status at the time of the first screening examination: oblast of residence (Zhytomyr, Kyiv, Chernihiv), type of residence (urban/rural), smoking status (yes/no), family history of thyroid disease (yes/no), presence of diffuse goiter on palpation (yes/no), and ultrasound-detected nodules (yes/no). Data also were cross-classified by sex, oblast of residence in 1986 (Zhytomyr, Kyiv, Chernihiv), and intake of iodine prophylaxis in May–June 1986 (yes/no). For each cross-classification cell, the number of observed thyroid cancers, PY, and PY-weighted means for continuous variables at the first screening examination (including TSH, ATPO, TG, thyroid volume, and urinary iodine) were computed.

We used Poisson regression models for grouped survival data to describe thyroid cancer incidence rates and to characterize radiation effects on these rates under relative and absolute risk models. Maximum likelihood parameter estimates, likelihood-based confidence intervals (CIs), and tests of independence or interaction were obtained using the AMFIT module of Epicure (Preston et al. 1993). Statistical tests were two sided at an α-level of 0.05.

The linear excess relative risk (ERR) model has the form *r*_0_(*x*)*(1 + β*dose), where *r*_0_ is the baseline or background incidence rate, *x* is the vector of covariates that describes the background rate, and β is the parameter that measures unit increase in the ERR per gray. The linear excess absolute risk (EAR) model has the form *r*_0_(*x*) + β*dose, where β is the absolute excess rate of thyroid cancer per gray that adds to the background thyroid cancer rate. In both ERR and EAR models, background rate was modeled as an exponential function of sex, oblast of residence at the first screening examination, and continuous attained age without interaction terms. The selected covariates were known to be major risk factors for thyroid cancer in various populations and/or influenced the magnitude of estimated radiation risk in preliminary analyses. The ERR and EAR are not nested models and cannot be compared directly; however, it is reasonable to suggest that the fit with lower deviance is the “better” one given the same degrees of freedom.

To test departure from linearity, we fitted linear-exponential [*r*_0_(*x*)*(1 + β*dose*exp^–γ*dose^) or *r*_0_(*x*) + β*dose*exp^–γ*dose^)] and linear- quadratic [*r*_0_(*x*)*(1 + β*dose + γ*dose^2^) or *r*_0_(*x*) + β*dose + γ*dose^2^)] models and compared their fit with the simple linear ERR and EAR models, respectively. A significant *p*-value at 1 degree of freedom (df) of likelihood ratio test comparing nested models indicates that the data were not consistent with linearity.

To test interaction or departure from the constant ERR or EAR models, we fitted the dose–response model with main effects only and compared its deviance with a model that also included dose–response parameters within *J* categories of factor of interest (sex, oblast, iodine prophylaxis, and diffuse goiter). A significant *p*-value at *J* – 1 df indicates that the effect of dose is not homogeneous across levels of the factor under consideration. Also, for continuous variables (age at exposure; attained age; time since exposure; and serum TG, TSH, and urinary iodine) we evaluated interaction with dose based on a 1 df test including an interaction term between continuous dose and factor under consideration.

## Results

*Background risk of thyroid cancer.* There were 65 incident thyroid cancer cases and 73,004 PY of observation in this study. Selected associations with background risk of thyroid cancer adjusted for I-131 dose based on the simple linear ERR models are summarized in Table 1. The associations with background factors based on the simple linear EAR models were similar (data not shown). The risk increased with attained age (*p* < 0.001), and the magnitude of this increase appeared similar in males and females (*p* interaction = 0.27; data not shown). Risk of thyroid cancer was four times higher in residents of Chernihiv oblast and > 2.5 times higher in residents of Kyiv oblast than in residents of Zhytomyr oblast at the first screening examination (*p* < 0.001). Also, larger thyroid volume (*p* = 0.06), presence of diffuse goiter (*p* = 0.11), ultrasound-detected nodules (*p* = 0.09), and elevated levels of serum TG (*p* = 0.01) at the first screening examination appeared to be associated with increased risk of thyroid cancer.

**Table 1:** RRs and 95% CIs for selected risk factors associated with background incidence of thyroid cancer.

Characteristic		PY (*n* = 73,004)		Cases (*n* = 65)		RR (95% CI)
Sex						
Male		35,240		28		1.00 (Referent)*a*
Female		37,764		37		1.34 (0.82–2.20)
*p*-Value for homogeneity						0.24
Attained age (years)*b*						
< 22		26,440		16		1.00 (Referent)*c*
22 to < 30		34,374		25		1.73 (0.90–3.33)
30 to < 40		12,190		24		5.10 (2.60–9.99)
*p*-Value for homogeneity						< 0.001
Oblast of residence*b*						
Zhytomyr		20,254*d*		11		1.00 (Referent)*e*
Kyiv		14,710		15		2.78 (1.24–6.22)
Chernihiv		37,988		39		4.12 (1.96–8.67)
*p*-Value for homogeneity						< 0.001
Type of residency*b*						
Rural		52,690*d*		43		1.00 (Referent)*f*
Urban		20,309		22		1.32 (0.76–2.28)
*p*-Value for homogeneity						0.33
Smoking status*b*^,g^						
Nonsmoker		50,718*d*		44		1.00 (Referent)*f*
Current smoker		22,279		21		1.12 (0.61–2.06)
*p*-Value for homogeneity						0.71
Family history of any thyroid disease*b*						
No		48,030		46		1.00 (Referent)*f*
Yes		7,426		7		0.97 (0.43–2.16)
*p*-Value for homogeneity						0.90
Unknown		17,547		12		0.80 (0.42–1.52)
Thyroid volume (mL)*b*						
< 8.0		16,868		15		1.00 (Referent)*f*
8.0 to < 12.0		29,795		18		0.61 (0.30–1.22)
12.0 to < 16.0		14,519		13		0.86 (0.40–1.85)
16.0–90.5		11,823		19		1.49 (0.72–3.04)
*p*-Value for homogeneity						0.06
Diffuse goiter*b*^,h^						
No		57,122		45		1.00 (Referent)*f*
Yes		15,882		20		1.55 (0.92–2.63)
*p*-Value for homogeneity						0.11
Characteristic		PY (*n* = 73,004)		Cases (*n* = 65)		RR (95% CI)
Ultrasound-detected nodule*b*						
No		71,335		60		1.00 (Referent)*f*
Yes		1,669		5		2.44 (0.96–6.19)
*p*-Value for homogeneity						0.09
Serum TG (ng/mL)*b*						
< 15		30,845		20		1.00 (Referent)*f*
15.0 to < 24.0		13,642		13		1.41 (0.70–2.84)
24.0 to < 60.0		18,503		17		1.35 (0.70–2.58)
60.0–643.0		4,649		12		3.78 (1.83–7.81)
*p*-Value for homogeneity						0.01
Unknown		5,363		3		1.42 (0.40–4.97)
Serum TSH (mIU/L)*b*						
< 1.26		18,443		14		1.00**(Referent)*f*
1.26 to < 1.80		16,684		14		1.13 (0.54–2.37)
1.80 to < 2.70		21,140		17		1.08 (0.53–2.19)
2.70–25.40		16,011		19		1.52 (0.76–3.04)
*p*-Value for homogeneity						0.60
Unknown		725		1		1.93 (0.25–14.79)
Urinary iodine (μg/L)*b*						
< 25.0		14,317		15		1.00 (Referent)*f*
25.0 to < 50.0		23,931		23		0.93 (0.48–1.78)
50.0 to < 70.0		12,519		11		0.82 (0.38–1.80)
70.0–750.3		15,634		11		0.63 (0.29–1.38)
*p*-Value for homogeneity						0.67
Unknown		6,604		5		0.64 (0.23–1.79)
Serum ATPO (U/mL)*b*						
< 10.0		25,165		20		1.00 (Referent)*f*
10.0 to < 25.0		19,668		17		1.08 (0.57–2.07)
25.0 to < 60.0		18,092		19		1.40 (0.75–2.63)
60.0–18066.0		9,347		8		1.00 (0.44–2.28)
*p*-Value for homogeneity						0.53
Unknown		732		1		1.82 (0.24–13.63)

**Figure 1:**
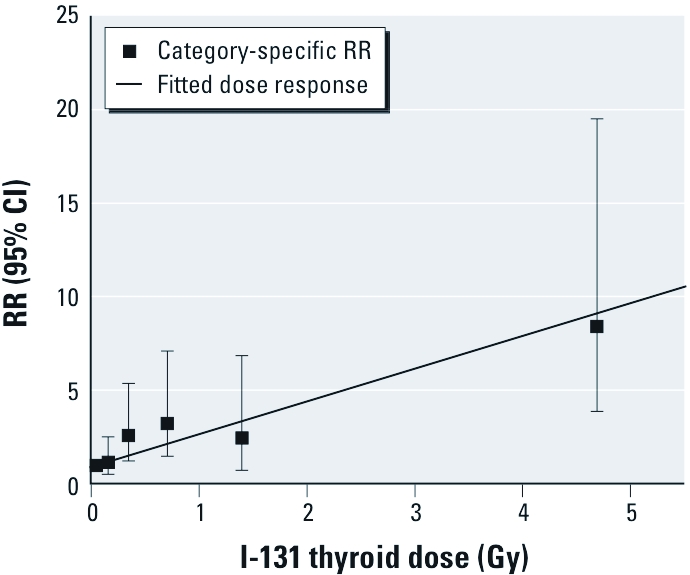
Dose–response relationship between incident thyroid cancers and I‑131 dose estimates in a cohort study of thyroid cancer and other thyroid diseases after the Chornobyl accident in Ukraine. The solid line represents fitted RRs based on the linear ERR model; data points and error bars represent category-specific RRs and 95% CIs for mean I‑131 dose per category. The fitted linear dose response was adjusted to pass through the lowest I‑131 category. The ERR was adjusted for sex, oblast of residence at the first screening examination, and continuous attained age.

*Dose response with I-131.* To evaluate the dose–response relationship with I-131, we fitted both linear ERR and EAR models. The estimated radiation risks were 1.91 (95% CI, 0.43–6.34; *p* < 0.001) for ERR per gray and 2.21 × 10^–4^ PY per gray (95% CI, 0.04 × 10^–4^ to 5.78 × 10^–4^; *p* = 0.02) for EAR, demonstrating a strong association with I-131 exposure for incident thyroid cancer. As evidenced from the models’ deviance, the linear ERR model appeared to describe our data better than did the linear EAR model (1001.799 vs. 1012.589, respectively). The I-131 dose-category–specific RRs and fitted RRs based on the simple linear ERR model are presented in Figure 1. The linear dose–response model provided an adequate fit to the categorical RRs. Inclusion of neither a linear-exponential (LE) nor a linear-quadratic (LQ) term significantly improved the fit compared with simple linear ERR (*p*_LE_ = 0.16 and *p*_LQ_ = 0.31) or linear EAR models (*p*_LE_ = 0.13 and *p*_LQ_ = 0.33).

When we repeated dose–response analyses for only papillary thyroid cancers (*n* = 61), the ERR per gray was 1.58 (95% CI, 0.30–5.44; *p* < 0.001) and the EAR per 10^4^ PY per gray was 1.71 (95% CI, < 0.06–4.95; *p* = 0.03). Exclusion of one incidentally found papillary thyroid microcarcinoma had no effect on either estimate of radiation risk (data not shown).

*Modification of the I-131 dose response.* The ERRs according to categories of selected factors are summarized in Table 2. The ERR per gray was higher for residents of Kyiv oblast and particularly of Chernihiv oblast than for residents of Zhytomyr oblast at the first screening examination (*p* interaction = 0.008). Similarly, the EAR in Kyiv was 5.78 (95% CI, 0.39–16.38), in Chernihiv 16.56 (95% CI, 7.83–27.92), and in Zhytomyr 0.37 (95% CI, < 0.01 to 2.27) per 10^4^ PY per gray (*p* interaction < 0.001). We observed the same pattern of radiation risks for place of residence in 1986 (*p* interaction for ERR = 0.005; *p* interaction for EAR < 0.001). The EAR, but not ERR per gray, varied significantly according to attained age, with older individuals having higher EARs (*p* interaction < 0.001). Specifically, EAR (per 10^4^ PY per gray) was 0.90 (95% CI, < 0.02–3.41) in individuals < 22 years of age; 5.25 (95% CI, 1.57–11.38), in 22- to < 30-year-olds; 5.25 (95% CI: 1.57–11.38), and 20.5 (95% CI, 6.24–42.08) in individuals ≥ 30 years of age. Although neither ERR nor EAR per gray varied significantly by other characteristics, some patterns are noteworthy. Specifically, the ERR per gray tended to increase with decreasing age at exposure and to be higher in females than in males; it was elevated throughout the observation period and did not vary meaningfully by time since exposure; and ERR per gray was somewhat higher for those who did not take iodine prophylaxis in 1986 or had diffuse goiter or higher levels of serum TG at the first screening examination. We observed no consistent pattern in radiation risk according to urinary iodine concentration or serum TSH at the first screening examination.

**Table 2:** Effect modification of the ERR of incident thyroid cancer per gray of exposure according to selected characteristics.

Table 2. Effect modification of the ERR of incident thyroid cancer per gray of exposure according to selected characteristics.						
Characteristic		PY (*n* = 73,004)		Cases (*n* = 65)		ERR per gray (95% CI)
Sex						
Male		35,240		28		1.20 (0.03 to 6.74)*a*
Female		37,764		37		2.66 (0.46 to 12.49)
*p*-Value*b*						0.40
Age at exposure (years)						
0 to < 4		21,236		18		7.43 (< 1.67 to NE)*c*
4 to < 12		34,941		31		1.57 (–0.02 to 8.51)
12 to < 18		16,827		16		0.69 (< –0.04 to 6.31)
*p*-Value						0.40
Attained age (years)						
< 22		26,440		16		2.08 (0.10 to > 13.65)*d*
22 to < 30		34,374		25		1.83 (0.01 to 11.97)
30 to < 40		12,190		24		0.74 (< 0.06 to 4.95)
*p*-Value						0.71
Time since exposure (years)*e*						
< 16.7		1,209		21		2.85 (0.08 to 44.31)*c*
16.7 to < 20.0		40,385		24		1.31 (0.05 to 7.18)
20.0–22.4		31,409		20		3.69 (0.43 to 42.80)
*p*-Value						0.60
Oblast of residence*f*						
Zhytomyr		20,254*g*		11		0.06 (< –0.02 to 1.08)*h*
Kyiv		14,710		15		2.70 (0.27 to 27.52)
Chernihiv		37,988		39		4.07 (0.95 to 16.80)
*p*-Value						0.008
Oblast of residence in 1986*f*						
Zhytomyr		20,816		12		0.08 (< –0.01 to 1.27)*h*
Kyiv		13,443		11		1.22 (–0.02 to 12.59)
Chernihiv		38,746		42		5.20 (1.45 to 21.40)
*p*-Value						0.005
Iodine prophylaxis in May–June 1986*f*						
No		51,674*g*		50*g*		2.11 (0.36 to 9.28)*c*
Yes		18,154		12		1.03 (< 0.08 to 9.84)
*p*-Value						0.56
Diffuse goiter*f,i*						
No		57,122		45		1.78 (0.33 to 6.41)*c*
Yes		15,882		20		2.07 (0.03 to > 28.4)
*p*-Value						0.89
Serum TG (ng/mL)*f*						
< 15.0		30,845*g*		20*g*		1.58 (< 0.32 to 13.77)*c*
15.0 to < 31.0		20,687		21		1.89 (0.18 to 12.21)
31.0–643.0		16,108		21		2.58 (0.16 to 34.73)
*p*-Value						0.92
Serum TSH (mIU/L)*f*						
< 1.3		17,265*g*		14*g*		2.61 (0.06 to 30.89)*c*
1.3 to < 2.5		33,750		28		1.97 (0.06 to 12.30)
2.5–25.4		19,448		21		1.05 (0.02 to 7.60)
*p*-Value						0.74
Urinary iodine (μg/L)*f*						
< 34.0		22,674*g*		20*g*		0.60 (< –0.002 to 5.69)*c*
34.0 to < 50.0		15,573		18		3.72 (0.36 to 52.48)
50.0–750.3		28,153		22		2.23 (0.16 to 18.49)
*p*-Value						0.36
NE, not estimable. **a**Adjusted for attained age and oblast. **b***p*-Value for tests of homogeneity of linear trends across categories of interest or 1 df interaction test between dose and continuous variable. **c**Adjusted for sex, attained age, and oblast. **d**Adjusted for sex, oblast, and time since exposure. **e**Difference between the exit date (date of thyroid surgery or last screening examination for those not operated upon) and date of the accident. **f**Reported at interview or detected during the first screening examination. **g**Person-years or cancer cases may not add up to column total because of missing values (excluded from analyses). **h**Adjusted for sex and attained age. **i**Based on palpation by endocrinologist and defined as grade 0, 1, or 2. In the analysis, grades 1 and 2 were combined as the “yes” category.						

Additional dose–response analyses according to pathomorphological size of the carcinoma categorized < 10 mm (*n* = 31) or ≥ 10 mm (*n* = 34) revealed a higher ERR per gray for the larger cancers (2.56; 95% CI, 0.46–11.26; *p* < 0.001) versus the smaller ones (0.90; 95% CI, < 0.05 to 8.00; *p* = 0.05), but this difference was not statistically significant (*p* = 0.97).

## Discussion

In this prospective cohort study of individuals exposed as children or adolescents to fallout from the Chornobyl accident, we found a significant dose–response relationship between individual I-131 thyroid dose estimates and risk of incident thyroid cancer 15–22 years after the accident. Like cohort studies of externally irradiated populations (Ron et al. 1995), our study found that the ERR model described the data better than the EAR model, and both models were consistent with linearity over the entire range of doses. The estimated ERR for incident thyroid cancer per gray was 1.91 (95% CI, 0.43–6.34), and the EAR was 2.21 × 10^–4^ PY per gray (95% CI, 0.04 × 10^–4^ to 5.78 × 10^–4^). The estimate of ERR per gray for incident thyroid cancer, although smaller, is compatible with the estimate of excess odds ratio per gray for prevalent thyroid cancer previously reported in this cohort (5.25; 95% CI, 1.70–27.5) (Tronko et al. 2006a) and in other case–control studies of post-Chornobyl thyroid cancer (Cardis et al. 2005; Davis et al. 2004). Similarly, the estimate of EAR in our cohort is comparable to the EARs observed in several post- Chornobyl ecological studies (Jacob et al. 1999, 2006; Likhtarov et al. 2006). The radiation risks from our study also could be compared with the risks from gamma rays and X rays reported in the pooled analysis of irradiated populations [ERR per gray = 7.7 (95% CI, 2.1–28.7) and EAR/10^4^ PY per gray = 4.4 (95% CI, 1.9–10.1), respectively] (Ron et al. 1995). Although the estimates from our study are lower than the respective estimates for gamma rays and X rays, some uncertainty remains in these comparisons because of wide CIs and lack of adjustment for age at exposure and other important confounders and/or effect modifiers.

Our results suggest that thyroid cancers attributable to I-131 exposure continue to occur two decades after exposure, with papillary thyroid cancer remaining the main histological type (94%). There is no indication of diminishing ERR per gray with increasing time since exposure within the narrow range of the available observation period. In a pooled analysis of thyroid cancer studies after external irradiation, excess risk peaked 15–19 years after exposure and then declined, although an excess was still apparent 40 years later (Ron et al. 1995). If studies of external radiation can serve as a guide, continued follow-up of this cohort is necessary to more accurately describe the pattern of radiation risks over time.

We found that the EAR, but not ERR per gray, increased with attained age, a finding that is likely to be related to increase in background rates of cancer with increasing age. One of the most consistent findings for thyroid cancer after external irradiation is a trend for increasing ERR per gray with younger age at exposure (Ron et al. 1995). This was also reported in previous studies of thyroid cancer after the Chornobyl accident (Jacob et al. 1999, 2006; Likhtarov et al. 2006; Tronko et al. 2006a). In the present study we demonstrated that the ERR per gray for 0 to < 4 years of age at exposure (7.43) was > 10 times that for 12 to < 18 years of age at exposure (0.69) (Table 2), although the trend was not statistically significant. In contrast to age at exposure, the variation of radiation risk by sex has been inconsistent (Cardis et al. 2005; Jacob et al. 1999, 2006; Likhtarov et al. 2006; Ron et al. 1995). In our data, the ERR and EAR per gray were higher in females, but the differences between males and females were not statistically significant.

Although the modification of I-131–related thyroid cancer risk by iodine prophylaxis or intake of stable iodine is important in view of the mild to moderate iodine deficiency prevailing in northern Ukraine (Nikiforova et al. 1997; Robbins et al. 2001; Tronko et al. 2005), it is difficult to study because data on iodine status at the time of the accident, the most relevant time period, are not readily available. In the only other analytical study that attempted to address this question, Cardis et al. (2005) found that self-reported intake of iodine prophylaxis was associated with lower risk of I-131–related thyroid cancer and that residence within iodine-deficient territories at the time of the accident was associated with higher risk of I-131–related thyroid cancer independent from each other. In the present study, individuals who reported intake of iodine prophylaxis had somewhat lower ERR per gray, although the difference was not statistically significant. Using a variety of indicators of stable iodine intake at the time of the first screening examination (i.e. 12–14 years after the accident), we found that the I-131–related risks tended to be higher among individuals with diffuse goiter or with higher levels of serum TG, but not in individuals with lower concentration of urinary iodine. Taken together, our data are not strong enough to support a modifying effect of either iodine prophylaxis or iodine deficiency on I-131–related risk of thyroid cancer, although our power to detect interactions was limited.

We found that background risk and I-131 excess risks exhibited significant variation according to oblast of residence at the first screening examination, with individuals from Chernihiv oblast, and to a lesser extent from Kyiv oblast, having higher risks than individuals from Zhytomyr oblast. Analyses using oblast of residence at the time of the accident provided similar results, reflecting the strong correlation between the two residences in time. Because screening of the cohort was conducted according to a standardized protocol and no meaningful differences were observed in compliance rates with FNAB or thyroid surgery by oblast, case ascertainment was unlikely to have caused a geographic difference. Interestingly, analyses of other thyroid outcomes in this cohort, including follicular adenoma (Zablotska et al. 2007), hypothyroidism (Ostroumova et al. 2009), and autoimmune-related outcomes (Tronko et al. 2006b), demonstrated that oblast of residence was also associated with background risk for these outcomes, suggesting a Zhytomyr–Kyiv–Chernihiv gradient. Thus, although three oblasts are generally similar in socioeconomic status and ethnicity, we cannot rule out a possibility that the oblast of residence may be a surrogate indicator for some unmeasured effect modifier. If oblast of residence was a surrogate marker for iodine deficiency status at the time of the accident and residents of Chernihiv oblast experienced stronger iodine deficiency in 1986 than did residents of Kyiv and Zhytomyr oblasts, this could have explained the observed pattern. Unfortunately, data concerning iodine status in the study areas in 1986 are not available, and 1991 data from the Sasakawa group (Nikiforova et al. 1997) are consistent with our data from 1998–2000 (Tronko et al. 2005) in that Zhytomyr oblast, but not Chernihiv oblast, appears generally more iodine deficient. Although we cannot explain the regional heterogeneity in the cohort, this does not change the fact that there clearly is a strong dose–response relation with I-131 exposure in the cohort.

One of the unresolved issues in studies of thyroid cancer after radiation exposure is the effect of screening on radiation risk estimates. Because everyone in our cohort was screened regardless of dose, confounding by screening intensity is unlikely. However, it remains controversial whether the detection of additional small thyroid cancers, which in the absence of thyroid screening might only have been detected later, would affect the excess radiation risks, as well as whether these small tumors are induced by radiation to the same extent as large tumors. The ERR per gray in the Michael Reese Hospital cohort of children externally irradiated for enlarged tonsils and adenoids was not significantly different before and after 1974, when the radiation issue became recognized and a screening program was initiated (Schneider et al. 1993). Similarly, in atomic bomb survivors Akiba et al. (1991) observed no significant difference in ERR per gray between those who were part of a periodically screened cohort and those who were not. Because the ERR appears to be unaffected by screening and represents a ratio of excess radiation cancers to background cancers, this implies that screening proportionally increases background and excess radiation cancers with small tumors and, therefore, that some of the small tumors are radiation related. In our cohort, direct evaluation of the dose response according to tumor size showed that, although the ERR per gray for cancers ≥ 10 mm was approximately three times larger than that for cancers < 10 mm, the ERR per gray for the small cancers was also elevated and the ERRs in both groups were not statistically different. The only other empirical data available on dose response by thyroid tumor size is from the cohort of atomic bomb survivors, in which radiation effects for small papillary carcinomas identified at autopsy were found (Hayashi et al. 2010; Sampson et al. 1969; Yoshimoto et al. 1995). Taken together, current evidence suggests that both small and large thyroid tumors are related to radiation exposure, yet additional data are necessary to determine if the magnitude of the dose response differs.

Although the main focus of our analysis was on quantification of I-131–related risk of incident thyroid cancer, our study also provides data on the epidemiology of thyroid cancer in general. Our findings of increased background risk of thyroid cancer among persons diagnosed with diffuse goiter or ultrasound-detected nodules at the first screening examination are consistent with findings from a pooled analysis of case–control studies of thyroid cancer in which a self-reported history of benign thyroid diseases was a significant thyroid cancer risk factor (Franceschi et al. 1999). Another interesting finding in our cohort was that the risk of thyroid cancer was approximately four times higher in those with serum levels of TG > 60 ng/mL compared with those with TG < 15 ng/mL. This association persisted after adjustment for presence of diffuse goiter or ultrasound-detected nodules at the first screening examination and is consistent with a previous report suggesting that serum TG, in addition to its established link with iodine deficiency, may be an early marker of thyroid cancer risk (Hrafnkelsson et al. 2000).

Among the major strengths of our study are its prospective nature, availability of individual I-131 dose estimates derived from radioactivity measurements taken within 2 months after the accident, low losses to follow-up (4.3%), near complete ascertainment of cases due to standardized screening examination, high rates of compliance with FNAB (75%) and surgery (80%), and review of pathological slides by an international panel of experts. A study limitation is the low statistical power to evaluate effect modification because of the small number of cases and the relatively short follow-up. We do not believe that the results of our study could be attributed to selection bias, even though during the first screening cycle we examined 40.9% of those originally selected for tracing or 67.5% of those located and invited to participate, because distribution of measured thyroid radioactivity was similar among participants (*n* = 13,243) and nonparticipants (*n* = 19,142) (Stezhko et al. 2004). The impact of uncertainties in dose estimates, 95% of which are typically attributable to unknown thyroid gland mass and the I-131 content in thyroid gland in 1986 (Likhtarev et al. 2003), was not taken into account. Because the error for thyroid mass is likely to be a mixture of classical error (which arises from an imprecise measuring device) and Berkson error (which arises when a single measurement is used to represent a group of subjects with varying true values), and because the error for direct thyroid measurement is largely classical, our study should have a mixed error structure. In a study of thyroid disease related to radiation fallout from the Nevada test site, Mallick et al. (2002) found that when a mixture error model was used to account for measurement error, radiation risk estimates were higher than unadjusted estimates, but < 100% higher than when all error was assumed to be classical. Based on these results, it seems reasonable to assume that the true radiation risk for incident thyroid cancer is likely to be higher than what we have reported, although with greater uncertainty.

## Conclusions

We found a significant linear dose–response relationship between individual I-131 thyroid dose estimates and risk of incident thyroid cancer two decades after the Chornobyl accident. The excess radiation risks, although somewhat smaller, were compatible with those of retrospective and ecological post-Chornobyl studies and studies of external irradiation and suggest that risk has not disappeared over time. However, additional follow-up of this cohort is necessary to more accurately describe the excess radiation risks by time since exposure and other factors.
